# Gaussian filter facilitated deep learning-based architecture for accurate and efficient liver tumor segmentation for radiation therapy

**DOI:** 10.3389/fonc.2024.1423774

**Published:** 2024-06-20

**Authors:** Hongyu Lin, Min Zhao, Lingling Zhu, Xi Pei, Haotian Wu, Lian Zhang, Ying Li

**Affiliations:** ^1^ Department of Oncology, First Hospital of Hebei Medical University, Shijiazhuang, China; ^2^ Technology Development Department, Anhui Wisdom Technology Co., Ltd., Hefei, China

**Keywords:** nnU-Net, Gaussian filter, liver tumor segmentation, deep learning, cyst

## Abstract

**Purpose:**

Addressing the challenges of unclear tumor boundaries and the confusion between cysts and tumors in liver tumor segmentation, this study aims to develop an auto-segmentation method utilizing Gaussian filter with the nnUNet architecture to effectively distinguish between tumors and cysts, enhancing the accuracy of liver tumor auto-segmentation.

**Methods:**

Firstly, 130 cases of liver tumorsegmentation challenge 2017 (LiTS2017) were used for training and validating nnU-Net-based auto-segmentation model. Then, 14 cases of 3D-IRCADb dataset and 25 liver cancer cases retrospectively collected in our hospital were used for testing. The dice similarity coefficient (DSC) was used to evaluate the accuracy of auto-segmentation model by comparing with manual contours.

**Results:**

The nnU-Net achieved an average DSC value of 0.86 for validation set (20 LiTS cases) and 0.82 for public testing set (14 3D-IRCADb cases). For clinical testing set, the standalone nnU-Net model achieved an average DSC value of 0.75, which increased to 0.81 after post-processing with the Gaussian filter (P<0.05), demonstrating its effectiveness in mitigating the influence of liver cysts on liver tumor segmentation.

**Conclusion:**

Experiments show that Gaussian filter is beneficial to improve the accuracy of liver tumor segmentation in clinic.

## 1 Introduction

Liver cancer is one of the most common malignant tumors worldwide (1). Radiotherapy, as one of the main treatment methods, plays a crucial role in liver cancer. Accurate delineation of liver tumor contours is essential for radiation oncologists and physicists to formulate precise treatment plans (2). However, manual delineation of liver tumors is time-consuming and labor-intensive, which may lead to errors due to the complexity of clinical practice, such as the variability of liver tumors in location, size and shape, low contrast between tumors and normal tissues, blurred lesion boundaries, and so on. Additionally, some low density tumors are often confused with liver cysts, requiring multiple imaging methods to distinguish cysts from tumors, posing a significant challenge for commonly used auto-segmentation algorithms. Therefore, accurate auto-segmentation of the liver tumor has become a challenging and valuable task.

Deep learning-based (DL) methods using convolutional neural networks (CNNs) have been widely used in medical image segmentation with excellent results (3–6), there are also previous studies of DL-based auto-segmentation for liver and liver tumors (7). In the public challenges of liver tumor segmentation from 2017 to 2019, deep learning methods demonstrated absolute advantages (8–18). Ben Cohen first applied fully convolutional networks (FCN) for liver segmentation and lesions detection, obtaining comparable performance relative to manual liver segmentation and detection with lower true positive rate (TPR) of 0.86 but better false positive per case (FPC) of 0.6 (9). Bellver et al. adjusted the deep retinal image understanding (DRIU) for detection aided liver lesion segmentation from computed tomography (CT) images (10). Han X. et al. used the modified 3D U-Net architecture to efficiently segment the liver and liver tumors, which used different skip connections and added multiple paths to extract multiple features. The model was trained with 130 LiTS training cases and achieved an average DSC of 0.67 for 70 testing cases, which ranked first in the ISBI 2017 annual conference but cannot be directly used in clinical practice yet (12). Alom et al. proposed RUNet and R2UNet for the detection of LiTS2017 datasets with high DSC of 0.75 (13). The AIM-Unet model proposed by Fırat Özcan (16) effectively solved the key flaw of U-Net’s inability to fully predict high-resolution edge information of advanced features in the input image, whose DSC values were 0.89 and 0.76, respectively. However, the segmentation results for the thin slice did not have advantages, with an average DSC of 0.68 on 3D-IRCADb dataset. In addition, the computational cost increased significantly for mU-Net. To solve the problem of high computational costs caused by a large amount of labeled data for 3D networks, the hybrid deep attention-aware network (RA-Unet) (17) and lightweight end-to-end S-Net that integrates spatial features and attention mechanism (18) were developed. RA-Unet achieved a DSC score of 0.59 for liver tumor segmentation in the LiTS2017 challenge dataset. S-Net can get high accuracy for large tumors with a Dice Global (DG) score of 0.78, but the segmentation results for small tumors are not good with a DG score of only 0.32. The challenge of segmenting small tumors still exists.

Various modified algorithms based on U-Net have made great progress in the auto-segmentation of liver tumors, and the testing results on standard datasets have been continuously improving. However, tumors in standard datasets often have regular shapes without much interference, whereas actual clinical liver tumor images contain uncertain factors such as cysts and calcifications. These factors increase the difficulty of liver tumor auto-segmentation in clinical practice. This study employed the nnU-Net (19, 20) model for liver tumor auto-segmentation and utilized Gaussian filter to analyze the HU distribution for post-processing. The segmentation results, with and without Gaussian filtering analysis, were compared and analyzed against manual delineations to verify the feasibility of our proposed method.

## 2 Materials and methods

### 2.1 Datasets

The Liver Tumor Segmentation Benchmark (LiTS) is a multi-center dataset (8), which contains 201 CT images of the abdomen, only 130 CT scans are made publicly available along with labels of liver tumor. Thus, the 130 cases of LiTS dataset are used for model training (110 cases) and validation (20 cases). For the model testing, 2 datasets are used to evaluate the accuracy of auto-segmentation model, including (1) 14 cases of the 3D-IRCADb dataset; (2) 25 clinical liver cancer cases retrospectively collected in our hospital.

There are 20 liver cases with ground truth in the 3D-IRCADb dataset (21), of which 14 cases have delineations of liver tumors, so 14 cases of the 3D-IRCADb dataset are used as the first testing dataset. For the second clinical testing datasets, 25 clinical cases were scanned on Philips Brilliance CT Big Bore large diameter CT machine for radiation therapy positioning at the First Hospital of Hebei Medical University. The scanning conditions were supine position with head first, with each layer having 512×512 pixels and layer thickness of 1.5 mm. The liver tumor target contours (Gross Tumor Volume, GTV) were delineated by experienced physicians using the Accuray PrecisionTM Treatment Planning System (Version 1.1.1.1) combined with magnetic resonance imaging (MRI) and positron emission tomography computed tomography (PET-CT).

### 2.2 Auto-segmentation model

**Figure 1:**
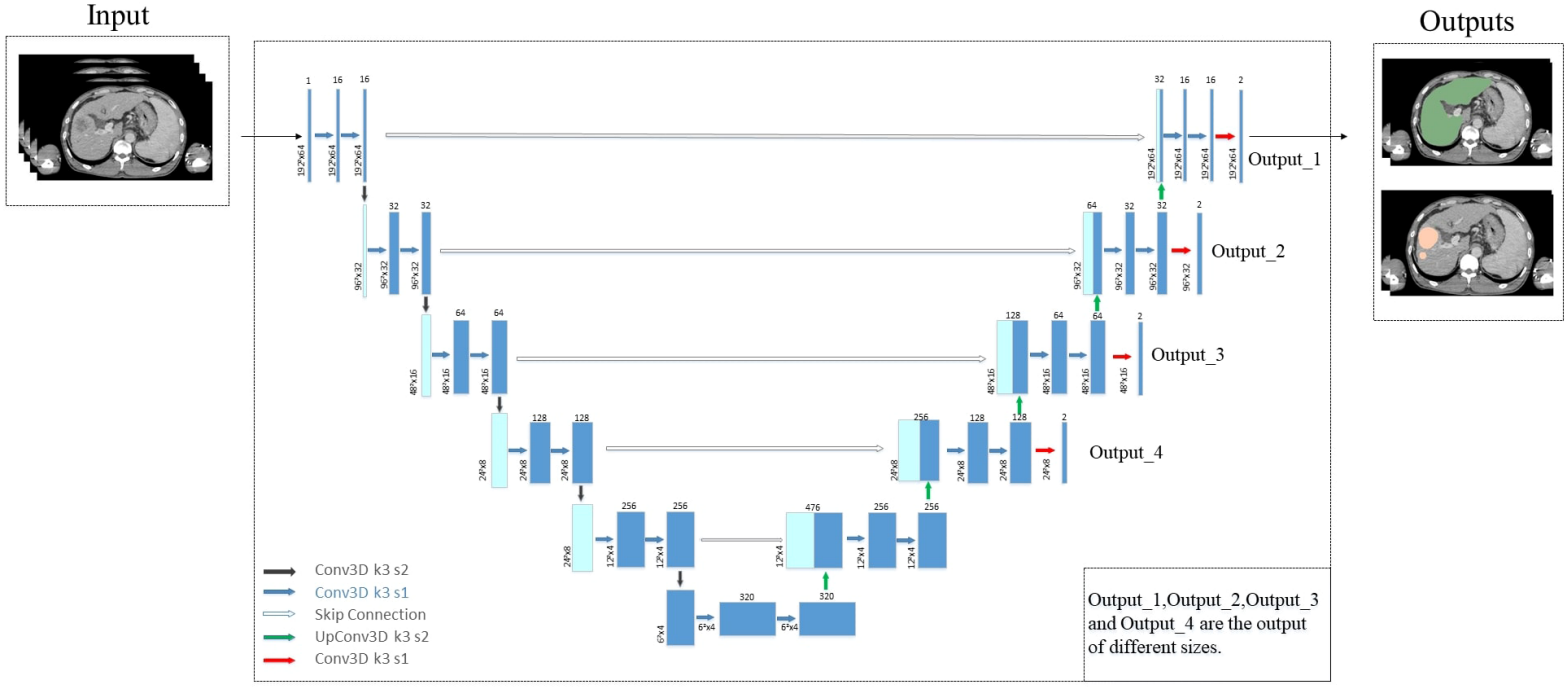
The architecture of 3D nnU-Net network.

The 3D nnU-Net auto-segmentation model architecture used in this study is shown in **Figure 1**. The network is based on the U-Net network with minor improvements, combining an encoder-decoder structure with skip connections. The network outputs result at four different scales and incorporates losses at different scales during backpropagation to improve the perception ability. Additionally, we found in actual clinical cases that many patients with liver cancer also have a large number of cysts. Since the HU values of liver tumors and cysts in CT images are very similar, the model cannot distinguish well between liver tumors and cysts. Therefore, we proposed a post-processing with Gaussian filter based on the HU values of cysts to screen out cysts and further improve accuracy of liver tumor segmentation.

### 2.3 Training strategy and improvements

The difficulty in liver cancer segmentation lies in the uncertainty of the tumor location, most tumors are on top of the liver. To improve accuracy and effectiveness, we use the 3D nnU-Net network to segment two structures, the liver tumor and the whole liver. This processing approach can restrict the tumor area within the liver rather than other parts of the body. Then, since the label of liver tumor is within the liver, the traditional Softmax activation layer can only output one probability distribution in the end, which can easily lead to problems with the segmentation accuracy of the overlapping areas for the tumor and the liver. To solve this problem, we use the Sigmoid activation method to obtain a probability distribution for liver tumor and the whole liver, which not only avoids the decreased accuracy in the overlapping areas but also limits the prediction range of the liver tumor area. Data preprocessing follows the nnU-Net framework, z-score normalization is used for data standardization, Gamma transformation and rotation is used for data augmentation.

The entire experimental process is implemented on Python 3.8, TensorFlow 2.6 and Windows 10, with an NVIDIA A10 graphics card. The specific parameter details are as follows: Dropout is set to 0.3, the number of epochs is set as 1000, the learning rate is 0.0002, and the optimizer is Adam.

### 2.4 Loss function design

Based on the training strategy designed above, the actual size of the whole liver differs significantly from the size of the liver tumor, so the losses should be calculated according to different scales. The liver tumor with small size is more sensitive to scale information, so multiscale outputs at different scales are used for backpropagation. The final loss function we designed is as Formula (1):


(1) $$\{\begin{array}{c} \text{Loss}=\sum_{\text{k}=1}^{\text{K}}\frac{1}{\text{K}}{\text{loss}}_{\text{k}}\\ {\text{loss}}_{\text{k}}=\frac{1}{2}\text{CrossEntropyLoss}+\frac{1}{2}\text{Diceloss}\\ \text{CrossEntropyLoss}=-\sum_{\text{c}=1}^{\text{C}}{\text{y}}_{\text{c}}\text{log}\left({\hat{\text{y}}}_{\text{c}}\right)\\ \text{DiceLoss}=1-\frac{1}{\text{C}}\sum_{\text{c}=1}^{\text{C}}\frac{2\left|\begin{array}{c} {\text{y}}_{\text{c}}\cap {\hat{\text{y}}}_{\text{c}} \end{array}\right|}{\left|{\text{y}}_{\text{c}}\right|+\left|{\hat{\text{y}}}_{\text{c}}\right|} \end{array}$$

where C is the number of classes (C=3 in this study: background, liver tumor and the whole liver), K is the total number of scales (K = 4), and the output scale is determined by the depth of the model. ${\text{y}}_{\text{c}}$ is the ground truth and ${\hat{\text{y}}}_{\text{c}}$ is the prediction. The single-scale loss function uses a weighted loss of cross-entropy and Dice loss, and the total loss function is the weighted sum of losses at different scales.

### 2.5 Gaussian filter

**Figure 2:**
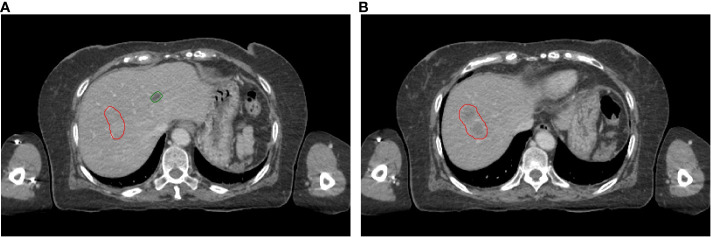
Visual display of liver tumors and cysts on slice 1 **(A)** and slice 2 **(B)**. Red lines: contours of liver tumors; Green lines: contours of cysts.

As shown in **Figure 2**, the HU values of cysts and liver cancer tumors are quite similar in CT images, it is difficult to distinguish them using CNN-based auto-segmentation model. In clinic, physicians manually delineated the contours of cysts and liver tumors based on the slight difference in HU values, with the help of functional images if necessary. To solve the problem automatically, we conducted statistical and analytical studies on the actual HU distributions of cysts and tumors, using their respective distribution characteristics for post-processing to distinguish cysts from liver tumors.

Gaussian filter is applied to fit the HU distributions of tumors and cysts, the goal of Gaussian fitting is to find the mean, standard deviation, and amplitude of the HU distribution data for tumors and cysts. The general form of the Gaussian distribution function can be described as Formula (2):


(2) $$f\left(HU\right)=\frac{A}{\sqrt{2\pi {\left(S\right)}^{2}}}e^{-\frac{{\left(HU-M\right)}^{2}}{2{\left(S\right)}^{2}}}$$

where HU is the independent variable; M is the mean of the Gaussian distribution; S is the standard deviation of the Gaussian distribution, representing the degree of dispersion; A is the amplitude.

**Figure 3:**
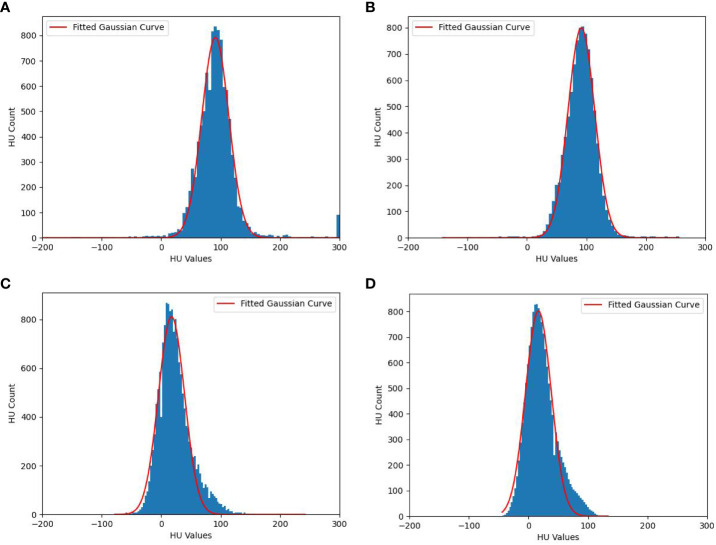
Fitted Gaussian curves of HU values for one random case. **(A)** Liver tumors without data smoothing, **(B)** Liver tumors with data smoothing, **(C)** cysts without data smoothing and **(D)** cysts with data smoothing.

There are noise points in the HU distributions, so the data have been smoothed before Gaussian filter to ensure the accuracy of the fitting. **Figure 3** shows the fitted Gaussian curves of HU values for tumors and cysts of a random case, the fitted Gaussian function basically describes the data distributions. **Figures 3A, C** shows the fitted Gaussian curves without data smoothing, **Figures 3B, D** shows the fitted Gaussian curves with data smoothing. The Gaussian parameters are A=799, M=91, and S=23 for liver tumors in **Figure 3B**, A=802, M=17, and S=22 for cysts in **Figure 3D**. Although **Figure 3** only shows a random patient, it reflects the difference in HU distributions between tumors and cysts, which provides a potential solution for removing cyst areas in auto-segmentation of liver tumors.

In summary, the HU distributions of cysts and tumors areas (GTV) both exhibit Gaussian-like distributions, which can be fitted with Gaussian distribution formulas. The amplitude, mean, and standard deviation can easily distinguish between cysts and GTV. As shown in **Figure 3**, the HU values of cysts are mainly distributed in the internal of [-20 HU, 80 HU], while the HU of tumors (GTV) is mainly distributed in the internal of [30 HU, 150 HU]. Based on this situation, we designed a post-processing process of liver tumor auto-segmentation. Firstly, the predicted auto-segmentation contours are divided into several independent regions, which may be tumors or cysts. Then, the Gaussian filter is used to fit the HU distribution of each independent region. Finally, the mean value of fitted Gaussian curve is used to distinguish between cysts and GTV. If the mean value is less than 45 HU, the region is considered as a cyst. On the contrary, it is the tumor area.

### 2.6 Evaluation metrics

The DSC and 95% Hausdorff distance (HD_95_) values were used to evaluate the accuracy of the auto-segmentation model. The DSC is defined as Formula (3):


(3) $$DSC=\frac{2\left|A\cap B\right|}{\left|A\right|+\left|B\right|}$$

where A denotes the auto-segmentation contours and B denotes the ground truth contours delineated by the physicians in our study. A larger DSC corresponds to a higher accuracy of auto-segmentation model. The DSC ranges from 0 to 1, with the latter value indicating perfect performance.

The HD is defined as Formulas (4, 5):


(4) HD(A,B)=max(h(A,B),h(B,A))

(5) $$\text{h}(A,B)=\underset{b\in B}{\max}(\underset{a\in A}{\min}\left\|a-b\right\|)$$

where h(A,B) is the greatest of all the distances from a point in A to the closest point in B. A smaller value usually represents better segmentation accuracy. The HD_95_ value represents the largest surface to surface separation among the closest 95% of surface points.

## 3 Results

**Figure 4:**
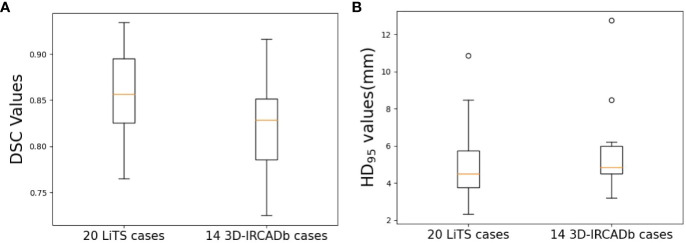
Boxplots obtained for DSC and HD_95_ analyses on publicly available datasets. **(A)** DSC; **(B)** HD_95_.

**Table 1** shows the results of nnU-Net used in this study for liver tumor auto-segmentation on publicly available datasets, the average DSC values are 0.86 and 0.82 for 20 LiTS cases and 14 3D-IRCADb cases, respectively. The min DSC value are both over 0.7, which is superior than some previous studies. The average HD_95_ values are 4.95 mm and 5.61 mm, some individual data have poor HD_95_ values, but overall the HD_95_ values are excellent. The boxplots obtained for DSC and HD_95_ analyses on publicly available datasets are displayed in **Figure 4**.

**Table 1:** Accuracy of nnU-Net for liver tumor auto-segmentation on publicly available datasets.

Datasets	Validation set (20 LiTS cases)		Testing set (14 3D-IRCADb cases)	
Evaluation matrix	DSC	HD_95_ (mm)	DSC	HD_95_ (mm)
Average value	0.86	4.95	0.82	5.61
Standard deviation	0.05	2.03	0.06	2.38
Min value	0.77	2.23	0.73	3.21
Middle value	0.86	4.51	0.84	4.84
Max value	0.93	10.85	0.92	12.74

Clinical liver cancer cases may have liver cysts, which will be confused with tumors. Physician oncologists rely on functional images (MRI or PET-CT) to accurately distinguish tumors and cysts manually. The public dataset only has labeled data for liver and liver tumors, without specifically labeling cysts. Therefore, directly applying the trained auto-segmentation model for clinical data can lead to cysts being mistaken for tumors, Gaussian filtering is used for post-processing in this study. As shown in **Table 2**, the DSC value of tumor contours directly predicted by the auto-segmentation model (Tumor_Pred) is 0.75 ± 0.07, and the HD_95_ value is 7.21 mm ± 2.43 mm. After the post-processing with Gaussian filtering (Tumor_Filtering), the DSC value could be increased to 0.81 ± 0.04 (P<0.05), and HD_95_ value is 5.25 mm ± 2.35 mm. According to the student t-test, the Tumor_Filtering contours are more accurate than the Tumor_Pred contours significantly (P<0.05).

**Table 2:** Clinical results of nnU-Net architecture using Gaussian filtering.

	DSC		HD_95_ (mm)	
cases	Tumor_Pred	Tumor_Filtering	Tumor_Pred	Tumor_Filtering
case 1	0.69	0.76	4.62	3.68
case 2	0.73	0.85	7.42	6.21
case 3	0.83	0.86	7.16	4.62
case 4	0.74	0.81	6.11	5.06
case 5	0.78	0.83	4.99	3.00
case 6	0.81	0.86	4.50	4.50
case 7	0.83	0.84	15.42	14.74
case 8	0.74	0.76	7.86	4.71
case 9	0.74	0.75	6.55	3.22
case 10	0.80	0.77	4.04	4.62
case 11	0.80	0.83	5.86	3.22
case 12	0.82	0.78	8.56	6.06
case 13	0.75	0.81	9.26	6.00
case 14	0.81	0.82	7.50	4.50
case 15	0.82	0.87	6.82	6.00
case 16	0.64	0.81	7.82	7.45
case 17	0.56	0.79	9.32	8.48
case 18	0.76	0.84	4.65	3.81
case 19	0.62	0.74	3.87	3.22
case 20	0.84	0.87	8.71	5.52
case 21	0.77	0.81	6.32	3.56
case 22	0.79	0.83	8.35	4.23
case 23	0.74	0.84	7.23	4.65
case 24	0.81	0.83	6.56	4.24
case 25	0.61	0.80	10.88	5.95
Ave ± Std	0.75 ± 0.07	0.81 ± 0.04	7.21 ± 2.43	5.25 ± 2.35
P* value	0.001		0.0006	

P was calculated by comparing the results of Tumor_Pred and Tumor_Filtering.

**Figure 5:**
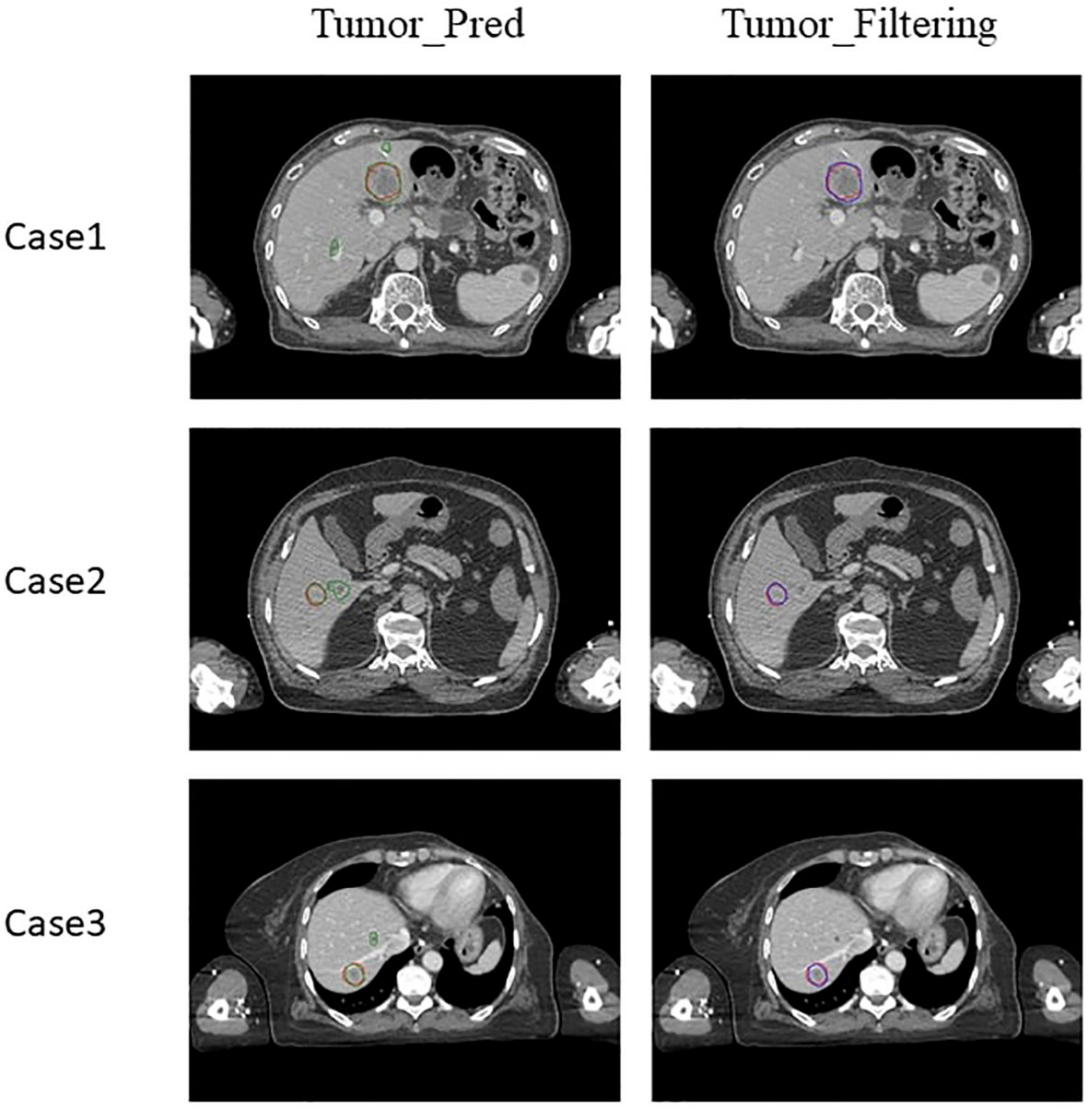
Liver tumor contour results of 3 clinical cases. Red lines: manual contours; Green lines: Tumor_Pred contours; Blue lines: Tumor_Filtering contours.

**Figure 5** shows three cases with Tumor_Pred contours (green lines) and Tumor_Filtering contours (blue lines), where the manual contours delineated by physician oncologists (red lines) are considered as ground truth. Overall, Gaussian filtering can effectively remove cysts and improve the efficiency of tumor identification and the accuracy of tumor auto-segmentation. For case 1 in **Figure 5**, there are two cyst regions around the tumor. Even though the HU value of the cyst visually appears to be significantly lower than that of the tumor, the auto-segmentation model still recognizes the cyst as the tumor, and the Gaussian filtering can effectively identify the cyst, increasing the DSC value from 0.75 to 0.81. The effectiveness of the Gaussian filtering method for identifying cysts is even more evident in case 2 (**Figure 5**), where tumors and cysts coexist in the liver, and the HU values of the tumor are like that of cyst, which is a significant challenge for liver tumor auto-segmentation. Gaussian filtering is still able to achieve excellent performance on the basis of auto-segmentation contours by nnU-Net, the DSC value can be improved from 0.80 to 0.83. The case 3 in **Figure 5** achieves the best performance among 25 testing cases in this study, the DSC values of Tumor_Pred contours and Tumor_Filtering contours are 0.82 and 0.87 respectively. The Tumor_Filtering contours can not only remove cysts, but also have a very high consistency with manually delineated contours, physician oncologists only need minor modifications to use it directly in clinic.

The implementation of Gaussian filtering is realized using Python scripts after obtaining the predicted auto-segmentation contours, which takes between 3 and 5 seconds, with an average time of 3.52 seconds. Because the auto-segmentation contours of the liver tumors are composed of multiple independent regions, it is necessary to process all regions and extract the HU values of the corresponding CT images for smoothing and Gaussian curve fitting when removing the cyst. Therefore, the cost time for Gaussian filtering depends on the number of regions in the auto-segmentation contours. The entire process is automated and does not require manual participation.

## 4 Discussion

CNN-based medical image segmentation method (22–24) has shown higher speed and accuracy compared to traditional medical image segmentation methods, such as Atlas-based auto-segmentation (25–27). This study implemented liver tumor auto-segmentation based on the nnU-Net architecture using Gaussian filtering, whose accuracy was validated on publicly available datasets and clinical datasets in our hospital. The auto-segmentation model achieved an average DSC value of 0.86 on 20 LiTS cases and 0.82 on 14 3D-IRCADb cases, the results proved that nnU-Net is excellent in the task of liver tumor auto-segmentation. To solve the problem of distinguishing between cysts and tumors in clinic, this study employed Gaussian filtering to effectively remove cysts from tumors, increasing average DSC value from 0.75 to 0.81. Effective identification of cysts can improve the quality of clinical planning. If cysts cannot be effectively removed, it will limit the realization of the maximum dose prescription and lead to the overdose of organs at risk. Take case 2 in **Figure 5** for example, if the larger cyst is mistaken for a tumor, high dose radiation will be given on this fake tumor during the planning process, which will increase the complexity of the radiotherapy plan and reduce the dose to the true tumors. This study combines the characteristics of clinical cases to achieve high precision liver tumor auto-segmentation, which is of great significance for improving the accuracy of clinical radiotherapy.

Public datasets provide a great platform for studying and comparing algorithm performance, but models trained on public datasets may suffer from accuracy loss when directly applied to clinical data. The average DSC of 20 LiTS cases and 14 3D-IRCADb cases are 0.86 and 0.82, which are slightly higher than the accuracy of the 25 clinical cases (0.81). This indicates that differences in cases, variations in conditions, and imaging of actual tumors under various modalities can all affect the delineation results of oncologists. As shown in **Figure 5**, the contours of auto-segmentation are strictly based on the HU distributions, while the manual contours are often experiential and preventive, so the manual contours will be slightly larger than the contours of auto-segmentation. This study incorporated expansion and corrosion to address this situation, in order to mimic the delineation habits of clinical oncologists as much as possible. In addition, the more prominent problem is that the shape of liver tumors is relatively regular, the tumor boundary density is relatively clear, and there is no distinction between liver tumors and liver cysts. This study provides a solution for new problems in liver tumor auto-segmentation, thereby improving the accuracy of auto-segmentation.

The generalization of deep learning models on different datasets is an inevitable issue. The cost of retraining the model is too high, and sufficient data is needed to achieve the expected accuracy. In this study, Gaussian filtering is a post-processing method based on image features, which effectively distinguishes tumors and cysts through Gaussian curve fitting. This is a processing method that does not require retraining of the model and is suitable for situations where the datasets to be applied are small. Previous researcher (28) has also proposed transfer learning methods based on pretrained models, which can achieve high accuracy with a small amount of training data. On the other hand, the consistency within the datasets is also an important factor limiting the accuracy of the auto-segmentation model. This study provides a preliminary proof of evaluating liver tumor auto-segmentation model trained on public datasets clinically, using Gaussian filtering with nnU-Net architecture could obtain excellent performance.

The auto-segmentation contours may have deviations, and the cyst area is relatively small. If only the average HU value within each independent region is simply counted, the deviation of the auto-segmentation contours will greatly affect the statistical results, which cannot effectively distinguish between tumors and cysts. In this study, the HU distributions of each region were smoothed and fitted by Gaussian filtering, which could avoid the influence of noise points. The HU values of cysts are mainly distributed lower than those of tumors, and they are all approximately convex distributions. Fortunately, the HU distributions of the tumors and cysts hardly overlap above 1% Percentage (99% points counted). As shown in **Figure 3**, 99% points of cysts fall into the interval of [9 HU, 50 HU], and 99% points of tumors fall into the interval of [67 HU, 112 HU]. Therefore, the HU distributions of cysts and tumors can be approximated and fitted with Gaussian curves, which can filter out cysts based on the actual HU distribution differences, thus improving the accuracy of auto-segmentation model.

Several limitations should be noted in this study. The boundaries of the tumors cannot be accurately identified in CT images, we will explore a multi-channel segmentation network combined with MRI or PET-CT images to improve the accuracy of liver tumor auto-segmentation. Additionally, we will collect more high-quality data and combine multiple methods to further improve the accuracy of the liver auto-segmentation model.

## 5 Conclusion

Liver tumor segmentation is a prerequisite step for diagnosis and treatment of liver cancer. However, clinical manual delineation of tumors is time-consuming and tedious. This study adopted the nnU-Net deep learning framework for liver tumor auto-segmentation and used Gaussian filtering to screen out false-positive cysts by the HU value distribution of cysts and tumors. This method eliminated the impact of liver cysts on the auto-segmentation of liver tumors, resulting in significant improvements in the evaluation of Dice index. To sum up, we proposed a new approach in the field of auto-segmentation of liver tumors, which could provide an effective reference for radiation oncologists to delineate liver tumors.

## Data availability statement

The raw data supporting the conclusions of this article will be made available by the authors, without undue reservation.

## Ethics statement

The studies involving humans were approved by Medical Ethics Committee of the First Hospital of Hebei Medical University. The studies were conducted in accordance with the local legislation and institutional requirements. The human samples used in this study were acquired from retrospectively collected clinical data. Written informed consent for participation was not required from the participants or the participants’ legal guardians/next of kin in accordance with the national legislation and institutional requirements. Written informed consent was obtained from the individual(s) for the publication of any potentially identifiable images or data included in this article.

## Author contributions

HL: Conceptualization, Methodology, Validation, Writing – original draft. MZ: Data curation, Resources, Writing – original draft. LZhu: Data curation, Writing – original draft. XP: Conceptualization, Supervision, Writing – review & editing. HW: Methodology, Writing – original draft. LZha: Conceptualization, Supervision, Writing – review & editing. YL: Conceptualization, Methodology, Supervision, Writing – review & editing.
